# 3Dmol.js: molecular visualization with WebGL

**DOI:** 10.1093/bioinformatics/btu829

**Published:** 2014-12-12

**Authors:** Nicholas Rego, David Koes

**Affiliations:** ^1^Department of Computational and Systems Biology, University of Pittsburgh, Pittsburgh, PA 15260, USA and ^2^Department of Biochemistry and Molecular Biophysics, University of Pennsylvania, Philadelphia, PA 19104, USA

## Abstract

**Summary:** 3Dmol.js is a modern, object-oriented JavaScript library that uses the latest web technologies to provide interactive, hardware-accelerated three-dimensional representations of molecular data without the need to install browser plugins or Java. 3Dmol.js provides a full featured API for developers as well as a straightforward declarative interface that lets users easily share and embed molecular data in websites.

**Availability and implementation:** 3Dmol.js is distributed under the permissive BSD open source license. Source code and documentation can be found at http://3Dmol.csb.pitt.edu

**Contact:** dkoes@pitt.edu

## 1 Introduction

Molecular visualization is an essential tool for computational chemists and biologists. Due to the demanding nature of three-dimensional (3D) graphics, most molecular viewers are desktop applications. The need to install specialized applications and, in some cases, the restrictive nature of the software licenses, introduces hurdles to the sharing of molecular data. Unlike a desktop application, a standards-based client-side web application comes pre-installed with every computer and mobile device with a modern web browser and can be seamlessly integrated into online environments for accessing and analyzing molecular data.

Currently, Jmol (Hanson, 2010) (http://www.jmol.org) is the most used web-based molecular viewer. Jmol is implemented as a Java applet and includes a custom rendering engine for efficiently rendering common molecular data representations, such as spheres and sticks. Due to this custom rendering engine and Java’s optimizing just-in-time compiler, the performance of Jmol can approach that of native, desktop applications. However, due to heavily publicized security failures, the Java install base is shrinking (Yegulalp, 2013). Even when Java is installed, users are presented with multiple security prompts that must be correctly navigated before a Java applet, such as Jmol, can run. To address these concerns, JSmol (Hanson *et* *al.*, 2013) was developed. JSmol is the product of applying a Java to JavaScript translator to Jmol. However, particularly for large and complex visualizations, the performance of JSmol lags behind that of Jmol.

An alternative to the software-based rendering of Jmol/JSmol is to use hardware-accelerated graphics, as is done with desktop applications. This is enabled by the recently adopted WebGL 1.0 standard, which is now supported natively by all major desktop and mobile browsers. PV (http://biasmv.github.io/pv) and GLmol (http://webglmol.sourceforge.jp) are two examples of WebGL-based molecular viewers. GLmol was the first WebGL viewer and uses the Three.js (http://threejs.org) framework for interfacing with WebGL. However, GLmol lacks a full featured API and the use of the Three.js library results in performance inefficiencies. We forked GLmol and radically reworked its architecture to overcome these deficiencies and create 3Dmol.js. PV, like 3Dmol.js, uses WebGL directly, but has a focus on displaying protein structures and does not provide the same set of features as 3Dmol.js.

## 2 3Dmol.js

**Fig. 1.**
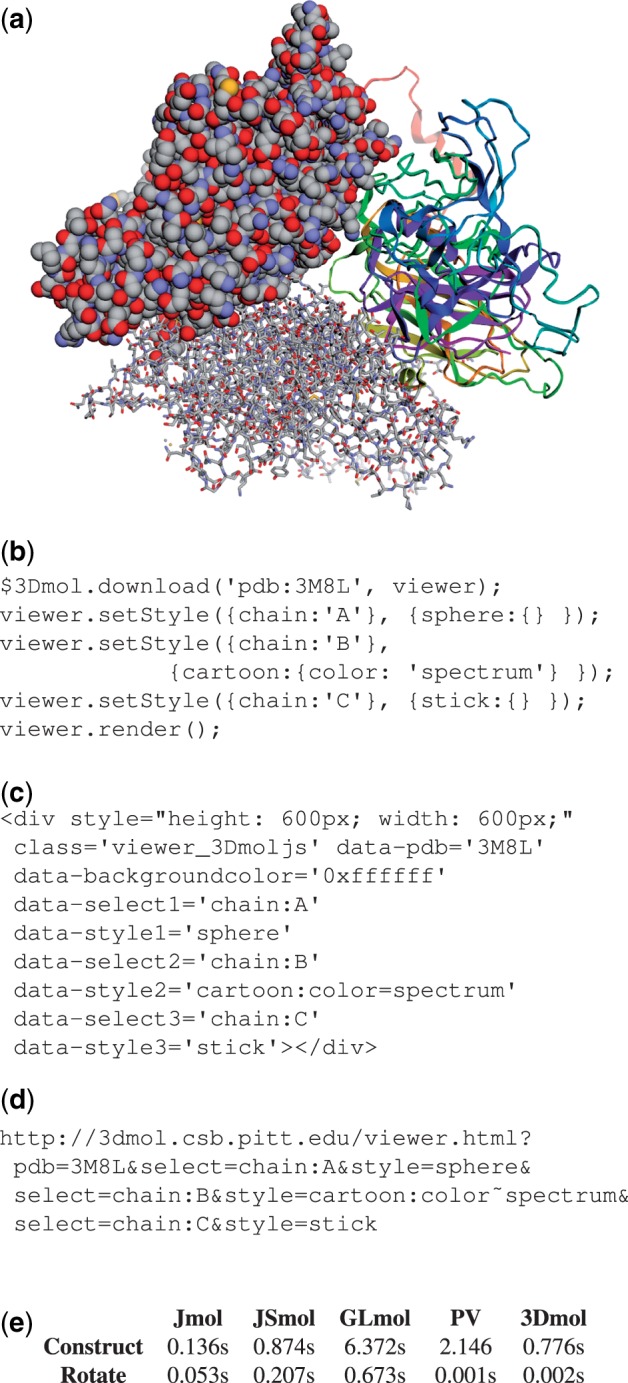
(**a**) A capsid protein (PDB: 3M8L) with 12 375 atoms as rendered by 3Dmol.js. This same scene can be generated (**b**) programmatically in JavaScript, (**c**) from within HTML or (**d**) by specifying a properly formatted URL to the 3Dmol.js hosted viewer. (**e**) The time required to create this scene and then rotate it for Jmol/JSmol 14.2.2, GLmol.47, PV v1.1-126-g85f16da and 3Dmol.js. PV was configured to be in high quality mode to better match the default quality of 3Dmol.js

3Dmol.js is a pure JavaScript, hardware-accelerated, object-oriented molecular visualization library that enables web developers and casual users to visualize and interact with molecular data in any modern desktop or mobile web browser with near native performance. The focus of 3Dmol.js is providing a full-featured API for online high-performance molecular visualization. This allows 3Dmol.js to be integrated with other web applications that provide additional cheminformatics and analysis capabilities. A variety of common styles are supported, as demonstrated by Figure 1a, and supported file formats include pdb, sdf, mol2, xyz and cube. 3Dmol.js can be used to view molecular data by web application developers, HTML authors and end users.

### 2.1 JavaScript API

JavaScript developers can use 3Dmol.js by including a single minified script and using the routines provided in the $3Dmol namespace. There are routines to manipulate and style molecular data, create isosurfaces from grid data, generate molecular surfaces, create arbitrary shapes, such as spheres and arrows, annotate the view with text and image labels and install callback handlers for when a user interacts with the viewer contents (e.g. clicks on an atom). Molecular styles include lines, crosses, sticks, spheres and cartoons, and atoms and surfaces can be colored by user specified properties, such as partial charge or atom type. An example of programmatically controlling a 3Dmol.js viewer to create the scene shown in Figure 1a is provided in Figure 1b.

### 2.2 Embeddable viewer

HTML authors do not need to use JavaScript to embed 3D viewers within their websites. 3Dmol.js will automatically turn any HTML element annotated with the viewer_3Dmoljs class into a viewer. The contents of the viewer are set and styled through the use of HTML data tags, as shown in Figure 1c. The molecular data can be retrieved from a remote URL or from an element that is embedded within the web page.

### 2.3 Hosted viewer

End users may use 3Dmol.js through a hosted viewer as shown in Figure 1d. In this case, the molecular data is set and styled through a URL specification. Data may be retrieved from a remote URL, such as a publicly accessible shared folder on cloud storage. This allows users to easily share complex scenes without requiring that the recipients have any software other than a modern web browser.

## 3 Performance comparison

The performance of 3Dmol.js is compared to Jmol, JSmol, GLmol and PV in Figure 1e. The time to create the scene of Figure 1a, which contains several visual styles applied to 12 375 atoms, and then to perform a single rotation was measured using JavaScript wall clock time. The scene was rendered in a 600 pixel square HTML element. Firefox 31 on a 2.4 GHz Core Duo 2008 MacBook with 4 GB of RAM running OS X 10.9.5 was used to time the operations and the average of the three best times of five trials is reported.

The initial creation time for a scene can be more time consuming in 3Dmol.js compared to a software-rendering approach like Jmol. The scene needs to be decomposed into a mesh of triangles since this is what is expected by the graphics subsystem. However, once a 3D scene is created, interactions with the scene that do not change its fundamental geometry, such as rotating, translating and zooming, are extremely fast (a few milliseconds) since the 3D scene data are managed by the native graphics subsystem. Consequently, even complex scenes can be smoothly manipulated by the user.

## 4 Conclusion

3Dmol.js is an high-performance interactive viewer for 3D molecular data that requires no plugins to work in modern desktop and mobile web browsers. 3Dmol.js provides a full-featured API to JavaScript developers, but can also be used by HTML authors and end users to share and distribute 3D visualizations of molecular data. 3Dmol.js is available under a permissive BSD open source license from http://3dmol.csb.pitt.edu.

## References

[btu829-B1] Hanson R.M. (2010) Jmol-a paradigm shift in crystallographic visualization. J. Appl. Crystallogr.*,* 43, 1250–1260.

[btu829-B2] Hanson R.M.*.* (2013) JSmol and the next-generation web-based representation of 3D molecular structure as applied to proteopedia. Isr. J. Chem.*,* 53, 207–216.

[btu829-B3] Yegulalp S. (2013) Java’s insecurity has doomed it on the desktop. InfoWorld*.* October 17.

